# The modified lymphocyte C-reactive protein score is a promising indicator for predicting 3-year mortality in elderly patients with intertrochanteric fractures

**DOI:** 10.1186/s12877-023-04065-z

**Published:** 2023-07-12

**Authors:** Zile He, Chuangxin Zhang, Mingzi Ran, Xin Deng, Zilin Wang, Yanhong Liu, Hao Li, Jingsheng Lou, Weidong Mi, Jiangbei Cao

**Affiliations:** 1 Department of Anesthesiology, The First Medical Center of Chinese PLA General Hospital, 28 Fuxing Road, Beijing, 100853 China; 2 Chinese PLA Medical School, Beijing, 100853 China; 3 Department of Anesthesiology, The Fourth Medical Center of Chinese PLA General Hospital, Beijing, 100037 China; 4 Department of Anesthesiology, Peking University People’s Hospital, Beijing, China; 5 Department of Liver Transplantation and Hepatobiliary Surgery, Shandong Provincial Hospital, Shandong First Medical University, Jinan, China

**Keywords:** Elderly patients, Intertrochanteric fractures, Indicator, Lymphocyte C-reactive protein score, Modified lymphocyte C-reactive protein score

## Abstract

**Background:**

Hip fractures are common in elderly patients, and almost all the patients undergo surgery. This study aimed to develop a novel modified lymphocyte C-reactive protein (CRP) score (mLCS) to simply and conveniently predict 3-year mortality in elderly patients undergoing intertrochanteric fracture surgery.

**Methods:**

A retrospective study was conducted on elderly patients who underwent intertrochanteric fracture surgery between January 2014 and December 2017. The mLCS was developed according to the value of CRP and lymphocyte counts. Univariate and multivariate Cox regression analyses were used to identify independent risk factors for 3-year mortality after surgery. The performances of the lymphocyte CRP score (LCS) and mLCS to predict 3-year mortality were then compared using C-statistics, decision curve analysis (DCA), net reclassification index (NRI) and integrated discrimination improvement (IDI).

**Results:**

A total of 291 patients were enrolled, of whom 52 (17.9%) died within 3 years after surgery. In the multivariate Cox regression analysis, mLCS (hazard ratio (HR), 5.415; 95% confidence interval (CI), 1.743–16.822; *P* = 0.003) was significantly associated with postoperative 3-year mortality. The C-statistics of LCS and mLCS for predicting 3-year mortality were 0.644 and 0.686, respectively. The NRI (mLCS vs. LCS, 0.018) and IDI (mLCS vs. LCS, 0.017) indicated that the mLCS performed better than the LCS. DCA also showed that mLCS had a higher clinical net benefit.

**Conclusions:**

mLCS is a promising predictor that can simply and conveniently predict 3-year mortality in elderly patients undergoing intertrochanteric fracture surgery.

**Supplementary Information:**

The online version contains supplementary material available at 10.1186/s12877-023-04065-z.

## Background

Hip fracture in senior people is a global problem with an increasing risk of morbidity [1] and mortality [2] and results in additional medical burdens [3]. By 2050, the number of hip fractures in elderly patients is expected to reach 4.5 million per year [1]. More than 40% of hip fractures are intertrochanteric fractures [4]. Several trials have been designed to assess 30-day mortality [5, 6] and 60-day mortality [4] following hip fracture surgery. However, studies evaluating risk factors for 3-year mortality are lacking.

Fresh light has been shed on the predictive potential of systemic inflammatory indicators for mortality, and these predictors include the platelet-to-lymphocyte ratio (PLR) [7], the C-reactive protein (CRP) [8], the neutrophil-to-lymphocyte ratio (NLR) [9], and the systemic immune-inflammation index (SII) [10]. In addition, multiple studies have shown that malnutrition is associated with increased mortality among geriatric patients with hip fractures [11]. Nonetheless, most indicators isolate the effect of inflammatory or nutritional variables [7, 9, 11, 12]. Recently, the lymphocyte-to-C-reactive protein (CRP) ratio (LCR) and score (LCS), which combine inflammation with the immune and nutrition status, have been widely used in many diseases and have achieved favourable predictive performance [13, 14]. However, the LCS has not been evaluated or applied to predict 3-year morbidity in elderly patients undergoing intertrochanteric fracture surgery.

In the present study, we conducted a retrospective study to evaluate the value of lymphocytes and CRP in predicting 3-year morbidity in elderly patients undergoing interventional surgery and to develop a novel mLCS indicator to conveniently and accurately predict 3-year morbidity in these patients.

## Methods

### Study design and patients

The study included patients who had all undergone proximal femur nail antirotation for intertrochanteric fractures at the Chinese PLA General Hospital between January 2014 and December 2017. The criteria for inclusion were as follows: patients were 65 years of age or more [10, 15] and underwent surgery for intertrochanteric fractures. The criteria for exclusion were as follows: patients had multiple fractures or trauma; had missing data or were lost to follow-up. A protocol for this study was approved by the Medical Ethics Committee of the Chinese PLA General Hospital (NO. S2019-311-02), and written informed consent was obtained from all patients for their data to be used for research purposes.

### Definition of variables and data Collection

Sex, age, mean arterial pressure (MAP), and smoking history were extracted from the admission records. A list of significant coexisting diseases was derived from the preoperative diagnostics in the medical records. The surgical conditions were collected from the anaesthesia records. Red blood cell (RBC) transfusions were obtained from the anaesthesia records and postoperative medical advice within 24 h. According to the discharge records, the length of hospitalization in the intensive care unit (ICU) was calculated.

Laboratory data, such as lymphocyte counts, CRP, haemoglobin, creatinine, D-dimer and albumin, were collected from the last time before surgery.

Okugawa *et al.* used the cut-off values for lymphocyte counts and CRP and developed a clinically feasible nutrition-inflammation marker known as the LCS to predict the outcomes in patients with gastric cancer [14]. The LCS indicator was developed according to each factor available prior to surgery [CRP ≤ 3.0 mg/L and lymphocyte counts ≥ 1 × 10^9^/L (0 points)]; [CRP > 3.0 mg/L or lymphocyte counts < 1 × 10^9^/L (1 point)]; [CRP > 3.0 mg/L and lymphocyte counts < 1 × 10^9^/L (2 points)].

In this study, we further adjusted the cut-off value of CRP using X-tile software and developed the mLCS scoring system. The detailed calculation method was as follows: patients with a lymphocyte count of ≥ 1 × 10^9^/L and a CRP level of ≤ 5.0 mg/L scored 0, patients with a lymphocyte count of < 1 × 10^9^/L or CRP > 5.0 mg/L scored 1, and patients with a lymphocyte count of < 1 × 10^9^/L and CRP > 5.0 mg/L scored 2.

### Endpoint

The endpoint of the study was 3-year mortality, defined as the proportion of patients who died within 3 years after surgery. The follow-up time was the time between surgery and the date of death or the last follow-up (December 31, 2020).

### Statistical analyses

Continuous variables that were normally distributed were expressed as the mean ± standard deviation (SD), while those that did not conform to the normal distribution were expressed as the median (interquartile range, IQR). Categorical variables were expressed as frequencies and percentiles. The appropriate cut-off value for CRP was determined using X-tile software. The survival curves were generated using the Kaplan‒Meier method, and the differences between the groups were compared using the log-rank test. Univariate and multivariate Cox regression analyses were used to identify independent risk factors for 3-year mortality. Receiver operating characteristic (ROC) curve analysis, decision curve analysis (DCA), net reclassification index (NRI) and integrated discrimination improvement (IDI) were used to assess the performance of different indicators in predicting 3-year mortality in these patients. All statistical analyses were conducted using two-sided analyses, and *P* < 0.05 was considered statistically significant. Data analysis was performed using SPSS software (IBM SPSS Statistics, version 25.0, Armonk, NY, USA), X-tile software (version 3.6.1, Yale University School of Medicine, New Haven, CT, USA), and R program (version 3.6.3, R Foundation for Statistical Computing, Vienna, Austria). The packages used in the R environment included “survival”, “rms”, “survminer”, “pROC”, “nricens”, “PredictABEL”, and “stdca”.

## Results

### Patient characteristics

**Fig. 1:**
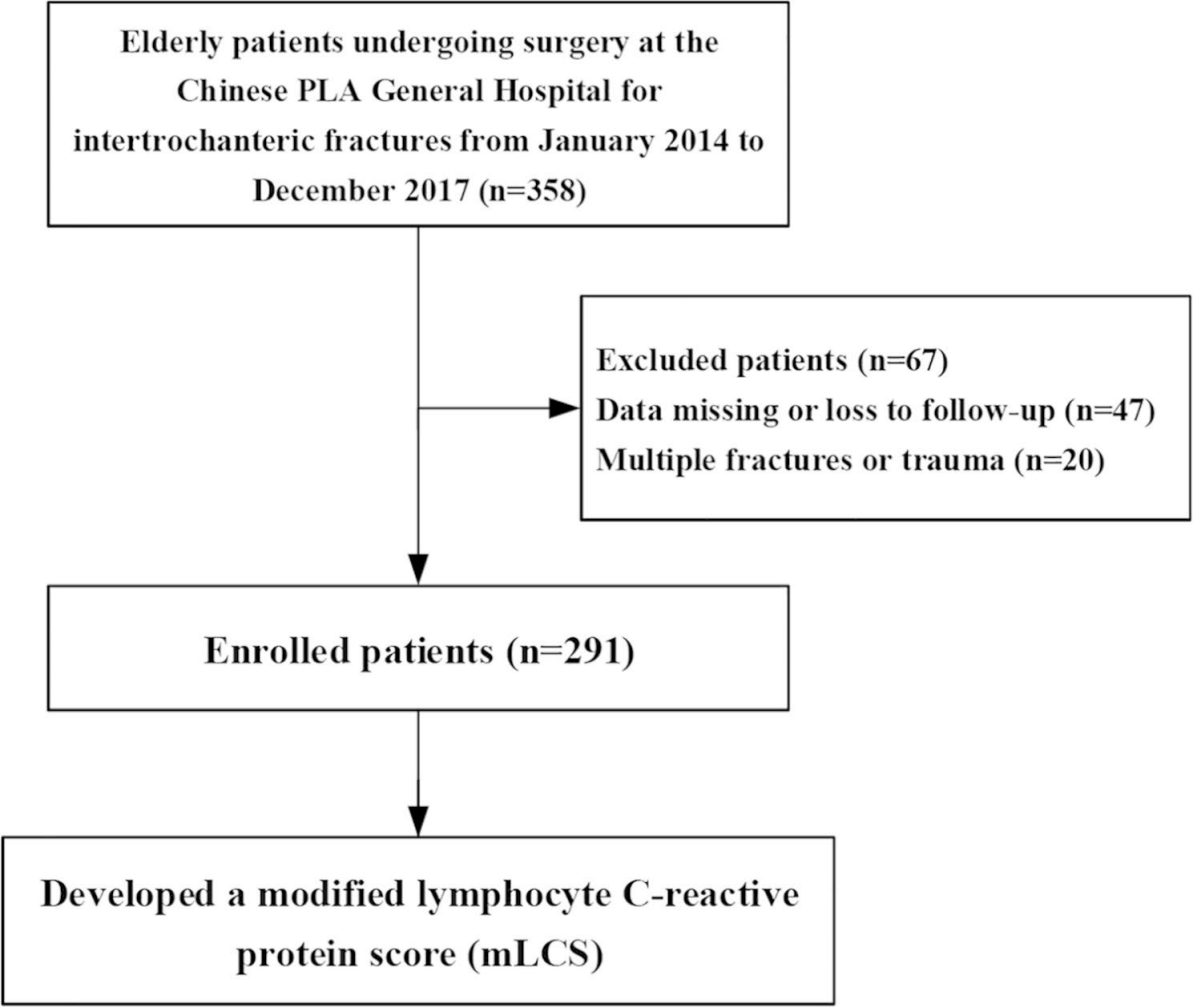
Flowchart of the patient selection process

From January 2014 to December 2017, we screened 358 patients for eligibility. Twenty patients suffered from multiple fractures or trauma. Forty-seven patients had missing data or were lost to follow-up. Because we did not have access to the final outcomes of these 47 patients during the follow-up period, we were unable to study the effect of mLCS on their 3-year mortality and had to exclude them. Eventually, a total of 291 patients were enrolled in the final analyses (Fig. 1).

Of the 291 patients, 75 (25.8%) were male, and 216 (74.2%) were female. The median age was 81.0 (76.0–86.0) years. The major coexisting conditions included pneumonia (26.5%), chronic obstructive pulmonary disease (COPD) (10.0%), diabetes mellitus (30.0%), hypertension (81.8%), and coronary artery disease (19.6%). For the surgical conditions, 203 (69.8%) patients received general anaesthesia, and 88 (30.2%) patients received regional or spinal anaesthesia. The median estimated blood loss was 100 (50–150) ml, and the median operative duration was 90.0 (70.0-105.0) minutes. After the operation, as some of the patients were older or had more comorbidities, 30 (10.3%) patients were admitted to the ICU for 3 days or more for safety, and 52 (17.9%) patients died within 3 years. The detailed baseline characteristics of all patients are shown in Table 1.

**Table 1:** The baseline characteristics of patients with intertrochanteric fractures (n = 291)

Variable	Characteristics †
Age, years	81.0 (76.0–86.0)
Sex	
Female	216 (74.2)
Male	75 (25.8)
BMI, kg/m^2^	22.8 ± 4.1
ASA stage	
I-II	131 (45.0)
> II	160 (55.0)
MAP, mmHg	97.3 (89.7-106.7)
Smoking history	28 (9.6)
**Major coexisting conditions**	
Pneumonia	77 (26.5)
COPD	29 (10.0)
Diabetes mellitus	87 (30.0)
Hypertension	238 (81.8)
Coronary artery disease	57 (19.6)
**Surgical conditions**	
Anesthesia method	
General anesthesia	203 (69.8)
Regional anesthesia	67 (23.0)
Spinal anesthesia	21 (7.2)
Estimated blood loss, ml	100.0 (50.0-150.0)
Operative duration, min	90.0 (70.0-105.0)
**Preoperative blood test**	
Hemoglobin, g/L	105.0 (96.0-115.0)
WBC, 10^9^/L	7.2 (5.7–8.9)
Creatinine, µmol/L	64.0 (52.9–80.8)
D-dimer, mg/L	3.0 (2.1–4.4)
Albumin, g/L	33.2 (31.2–35.3)
Total protein, g/L	62.8 ± 6.0
CRP, mg/L	3.1 (1.7-5.0)
Lymphocyte, 10^9^/L	1.2 (0.9–1.6)
**Postoperative conditions**	
Postoperative ICU ≥ 3 days	30.0 (10.3)
Died within 36 months	52 (17.9)

†, Data are presented as n (%) or mean ± SD or median (IQR).

**Abbreviations**: ASA, American Society of Anesthesiologists; SD, standard deviation; IQR, interquartile range; MAP, mean arterial pressure; COPD, chronic obstructive pulmonary disease; WBC, white blood cell; CRP, C-reactive protein; ICU, intensive care unit

### Identification of independent prognostic factors for 3-year Mortality in Elderly patients with intertrochanteric fractures

The univariate Cox regression analysis showed that age (hazard ratio [HR] 1.094; 95% confidence interval [CI] 1.050–1.141; *P* < 0.001), American Society of Anaesthesiologists (ASA) stage > II (HR 2.392; 95% CI 1.296–4.414; *P* = 0.005), mean arterial pressure (MAP) (HR 0.974; 95% CI 0.953–0.996; *P* = 0.021), pneumonia, (HR 1.890; 95% CI 1.081–3.305; *P* = 0.026), haemoglobin (HR 0.977; 95% CI 0.956–0.998; *P* = 0.034), creatinine (HR 1.014; 95% CI 1.007–1.020; *P <* 0.001), preoperative LCS > 1 (HR 2.228; 95% CI 1.267–3.919; *P* = 0.005), preoperative mLCS > 1 (HR 4.310; 95% CI 2.362–7.866; *P* < 0.001), and postoperative ICU ≥ 3 days (HR 3.161; 95% CI 1.658–6.026; *P <* 0.001) were associated with postoperative 3-year mortality (Table 2). The multivariate analysis demonstrated that age (HR 1.069; 95% CI 1.019–1.121; *P* = 0.006), creatinine (HR 1.011; 95% CI 1.004–1.018; *P* = 0.003), mLCS (HR 5.415; 95% CI 1.743–16.822; *P* = 0.003) and postoperative ICU ≥ 3 days (HR 3.145; 95% CI 1.582–6.255; *P =* 0.001) were independent prognostic factors for postoperative 3-year mortality (Table 2).

**Table 2:** Univariate and Multivariate Cox Analysis for 3-year Mortality in Elderly Patients with Intertrochanteric Fractures

Variables	Univariate analysis					Multivariate analysis			
	B	HR	95% CI	*P* value		B	HR	95% CI	*P* value
Age, per year	0.090	1.094	1.050–1.141	< 0.001*		0.067	1.069	1.019–1.121	0.006*
Sex, male vs. female	0.043	1.044	0.566–1.927	0.890					
ASA stage, > II vs. I-II	0.872	2.392	1.296–4.414	0.005*		0.271	1.311	0.675–2.549	0.424
BMI, > 24 vs. ≤ 24, kg/m^2^	-0.547	0.579	0.313–1.068	0.080					
MAP, per mmHg	-0.026	0.974	0.953–0.996	0.021*		-0.018	0.982	0.960–1.005	0.118
Smoking history, yes vs. no	0.588	1.801	0.848–3.826	0.126					
**Major coexisting conditions**									
Pneumonia, yes vs. no	0.637	1.890	1.081–3.305	0.026*		0.457	1.579	0.882–2.828	0.124
COPD, yes vs. no	0.145	1.157	0.494–2.708	0.738					
Diabetes mellitus, yes vs. no	-0.397	0.673	0.353–1.282	0.228					
Hypertension, yes vs. no	-0.564	0.569	0.308–1.050	0.071					
Coronary artery disease, yes vs. no	-0.682	0.506	0.216–1.184	0.116					
**Surgical conditions**									
Anesthesia method									
Regional vs. general	0.245	1.277	0.687–2.373	0.439					
Spinal vs. general	-0.197	0.821	0.253–2.670	0.743					
Estimated blood loss, per ml	-0.004	0.996	0.992-1.000	0.075					
Transfusion, yes vs. no	3.139	23.077	0.329-1616.745	0.148					
**Preoperative blood test**									
Hemoglobin, per g/L	-0.024	0.977	0.956–0.998	0.034*		-0.016	0.984	0.962–1.006	0.162
WBC, per 10^9/L	0.017	1.017	0.903–1.145	0.781					
Creatinine, per µmol/L	0.014	1.014	1.007–1.020	< 0.001*		0.011	1.011	1.004–1.018	0.003*
D-dimer, per mg/L	-0.006	0.994	0.889–1.110	0.909					
Albumin, per g/L	-0.060	0.941	0.864–1.025	0.167					
Preoperative LCS, > 1 vs. ≤ 1	0.801	2.228	1.267–3.919	0.005*		-0.455	0.634	0.220–1.827	0.399
Preoperative mLCS, > 1 vs. ≤ 1	1.461	4.310	2.362–7.866	< 0.001*		1.689	5.415	1.743–16.822	0.003*
**Postoperative conditions**									
Postoperative ICU ≥ 3 days, yes vs. no	1.151	3.161	1.658–6.026	< 0.001*		1.146	3.145	1.582–6.255	0.001*

* These variables were statistically significant in univariable or multivariable analysis (*P* < 0.05)

**Abbreviations**: B, coefficient; HR, Hazard Ratio. CI, Confidence interval; MAP, mean arterial pressure; COPD, chronic obstructive pulmonary disease; ASA, American Society of Anesthesiologists; WBC, white blood cell; LCS, lymphocyte CRP score; mLCS, modified lymphocyte CRP score; CRP, C-reactive protein; ICU, Intensive Care Unit

### Survival analysis and discrimination for LCS and mLCS stratified by different risk groups

**Fig. 2:**
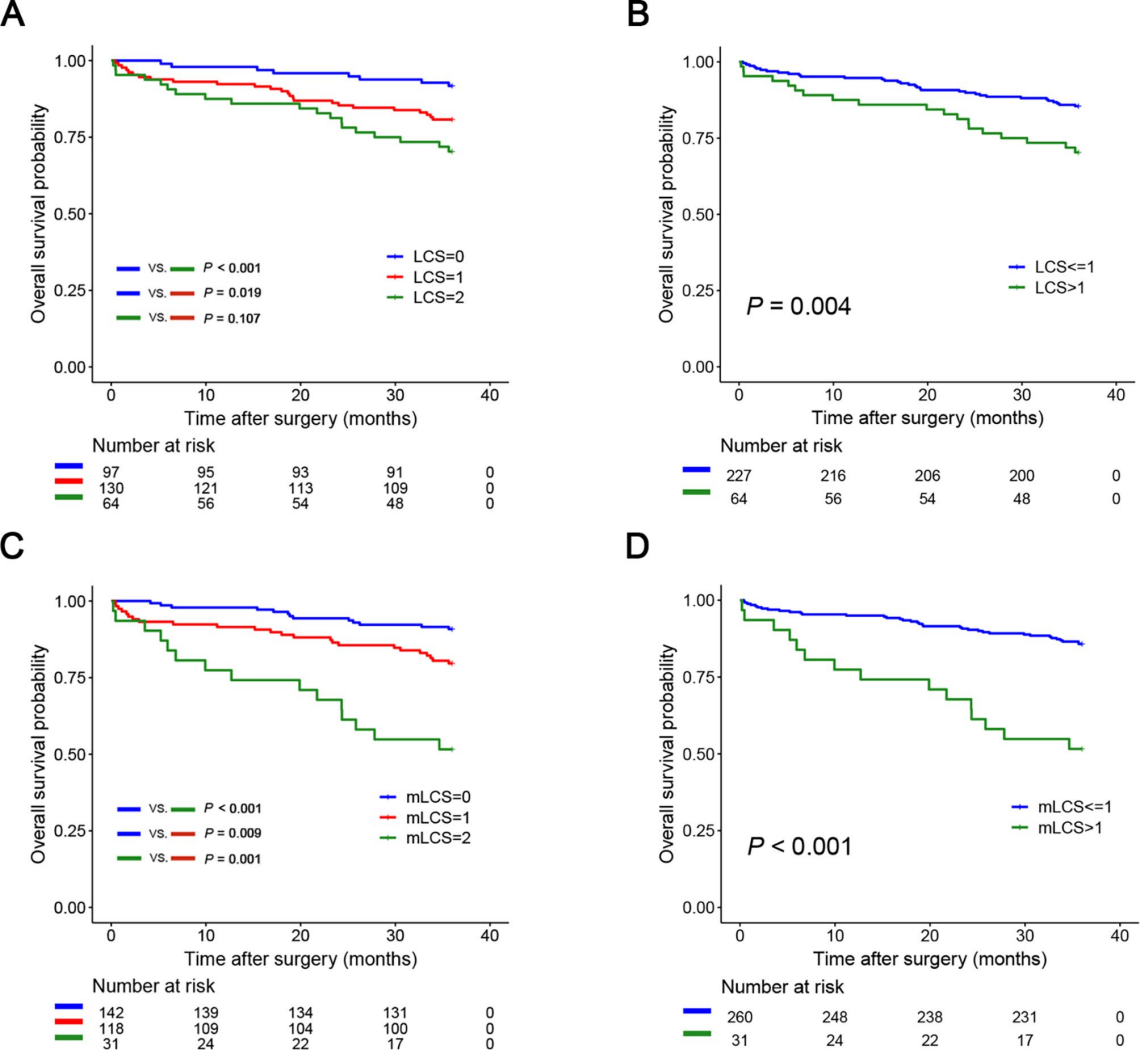
Kaplan‒Meier curves estimating overall survival (OS) according to the different LCS and mLCS groups. (A, survival outcomes of patients in the LCS = 0, 1, 2 groups; B, survival outcomes of patients in the LCS > 1 and LCS ≤ 1 groups; C, survival outcomes of patients in the mLCS = 0, 1, 2 groups; D, survival outcomes of patients in the mLCS > 1 and mLCS ≤ 1 groups). LCS, lymphocyte C-reactive protein score; mLCS, modified lymphocyte C-reactive protein score

As shown in Fig. 2A and B, although the patients with an LCS ≤ 1 had a significantly better prognosis than those with an LCS > 1 (*P* = 0.004), the LCS indicator was not good at distinguishing the long-term survival outcomes of the patients with scores of 1 and 2 (*P* = 0.107). According to the novel mLCS metric, there were significant differences in the long-term survival outcomes of the patients with different scores (mLCS = 0 vs. mLCS = 1, *P* = 0.009; mLCS = 0 vs. mLCS = 2, *P* < 0.001; mLCS = 1 vs. mLCS = 2, *P* = 0.001; mLCS > 1 vs. mLCS ≤ 1, *P* < 0.001) (Fig. 2C and D). The detailed long-term survival outcomes for the patients in the different risk groups are shown in Supplementary Tables 1 and Supplementary Table 2.

**Fig. 3:**
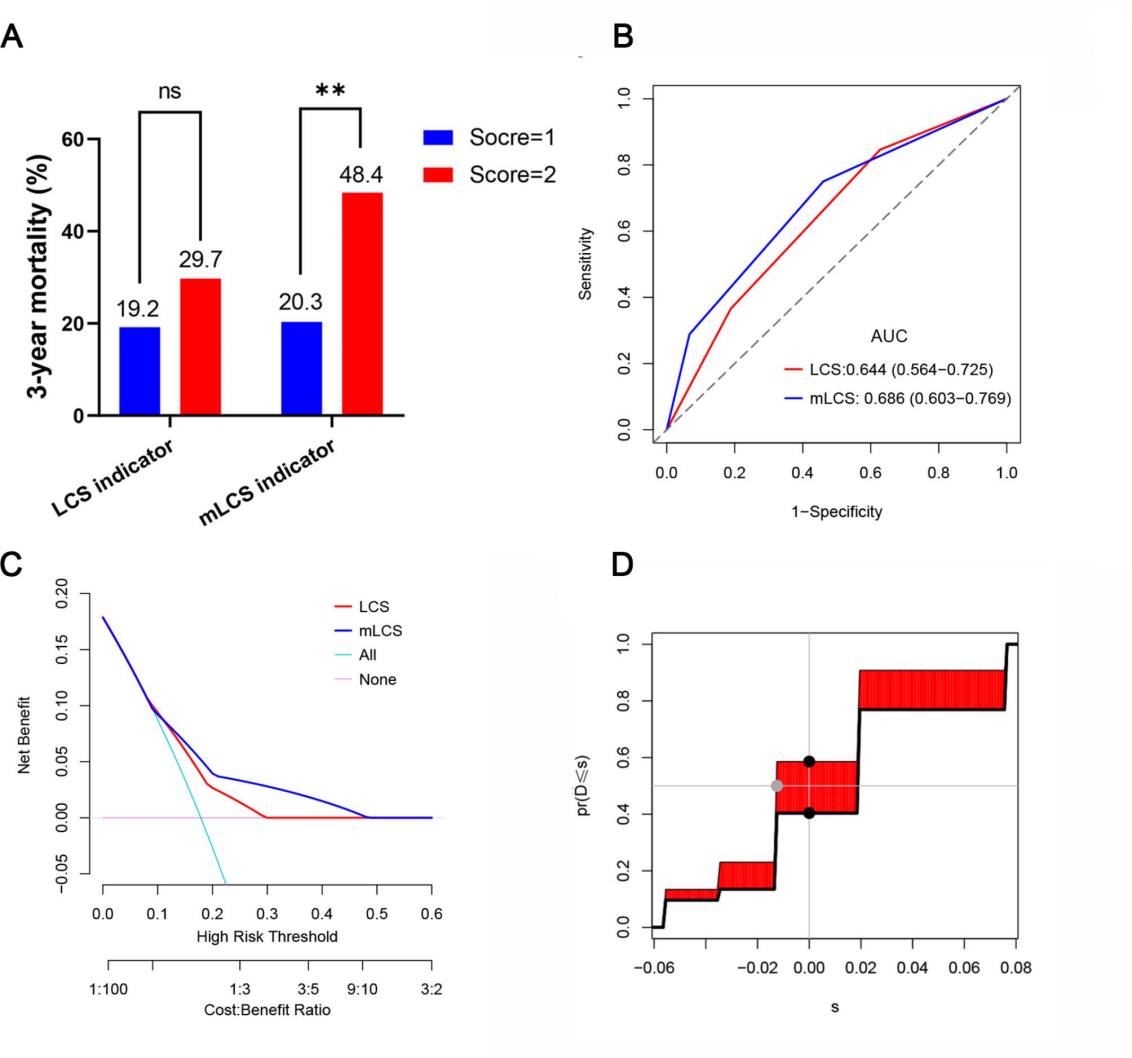
Comparison of discrimination, predictive accuracy and clinical usefulness of LCS and mLCS for 3-year mortality in elderly patients with intertrochanteric fractures. (A, comparison of discrimination between LCS and mLCS; B, ROC curves showing the performance of the two indicators in predicting 3-year mortality; C, DCA analysis showing the net benefits of the two indicators in predicting 3-year mortality; D, graphical representation of IDI). ROC, receiver operating characteristic; DCA, decision curve analysis

When the mLCS = 2, the 3-year mortality was 48.4%, which was significantly higher than that of the patients with mLCS = 1 (48.4% vs. 20.3%, *P* = 0.002), while the patients with LCS = 1 and 2 had no significant difference in 3-year mortality (29.7% vs. 19.2%, *P* = 0.102) (Fig. 3A). See Supplementary Table 3 for detailed results on postoperative 3-year mortality for LCS and mLCS in the patients within the different risk groups.

### Comparison of the predictive accuracy and clinical usefulness of the LCS and mLCS for 3-year mortality in elderly patients with intertrochanteric fractures

The C-statistics of LCS and mLCS for predicting postoperative 3-year mortality were 0.644 (95% CI: 0.564–0.725) and 0.686 (95% CI: 0.603–0.769), respectively. The detailed results are shown in Supplementary Tables 4 and Fig. 3B.

Decision curve analysis (DCA) converts a complex mathematical model into a simple graph to intuitively judge the net benefits of different indicators for predicting postoperative 3-year mortality [16]. DCA revealed that the mLCS provided superior clinical net benefits when compared with LCS within the threshold range of 0.1–0.5 (Fig. 3C).

NRI and IDI were used to further demonstrate the improved prediction performance of the novel indicator compared to the old indicator [17]. As shown in Supplementary Tables 5 and Fig. 3D, the NRI and IDI of the mLCS compared to the LCS in predicting 3-year mortality in elderly patients with intertrochanteric fractures were 0.018 (95% CI: -0.051-0.063) and 0.017 (95% CI: -0.003-0.054), respectively.

## Discussion

In this retrospective, observational study involving 291 elderly patients undergoing intertrochanteric fracture surgery, we evaluated the predictive efficacy of LCS and mLCS in assessing postoperative 3-year mortality.

Multiple studies published in the last few years have demonstrated that different inflammatory variables can serve as prognostic indicators for postoperative outcomes among patients with malignancies, and these indicators include PLR [7], CRP [8], NLR [9] and SII [10]. However, these indicators primarily focus on the impact of inflammatory or nutritional factors separately. In recent years, LCR and LCS, which combine inflammation with immune and nutritional status, have been widely used in the research of many diseases and have achieved favourable prediction performance [13, 14]. Serum levels of CRP are associated with the systemic inflammatory response, and CRP has been shown to play a role in host defence and inflammation [18, 19]. An increasing body of evidence indicates that patients with high CRP levels have a significantly worse prognosis [8, 20–24]. Peripheral lymphocytes are primarily composed of T cells, B cells, and NK cells. These cells are essential for the host’s immune response. Among COVID-19 and haematologic cancer patients, Bange *et al.* [25] proposed that CD8 (+) T cells play an important role in the survival of patients. He thought that an increase in CD8 (+) T cells would increase survival rates. B cells contribute to normal immune responses in ways beyond the ability to produce antibodies [26]. Petitprez *et al.* [27] found that B cells may influence immunity and contribute to survival in sarcoma patients. The B-cell-high group was found to have a higher survival rate. Moreover, peripheral lymphocyte counts are a critical component of nutritional indices, such as CONUT (The Controlling Nutritional Status score) and PNI (The Prognostic Nutritional Index score), which were previously used to evaluate malnutrition. An increased risk of death was associated with malnutrition compared with good nutrition [28, 29]. In addition, Price *et al.* [30] observed that the absolute lymphocyte count predicted overall survival in oropharyngeal squamous cell cancer patients. In summary, low lymphocyte counts indicate poor overall survival.

In light of these findings, high CRP levels and low lymphocyte counts may represent a poor immune response in the host, increased systemic inflammatory conditions, and malnutrition. Notably, we modified the existing scoring system (LCS) and introduced a new system (mLCS). The results of this study indicated that a higher mLCS score was an independent predictor for 3-year mortality among patients with intertrochanteric fractures, which is consistent with previous research [28–30]. Despite this, LCS did not show a significant effect on 3-year mortality after surgery. In addition, we further validated the predictive accuracy, discriminative power, and net clinical benefit of these two metrics in predicting 3-year postoperative mortality in these patients. The results revealed that the mLCS developed in this study exhibited a better prediction performance. Hopefully, the mLCS may prove to be a simple, promising and valuable indicator for predicting 3-year postoperative mortality in elderly patients following intertrochanteric fracture surgery. Furthermore, Survival analysis showed that patients with an mLCS ≤ 1 (low-risk) had a significantly better long-term prognosis than those with an mLCS > 1 (high-risk). Since both lymphocyte counts and CRP levels can be obtained pre-operatively, clinicians may use more aggressive treatment and follow-up strategies to improve long-term outcomes in high-risk patients. Of course, further research is needed to confirm the clinical significance of mLCS.

There are several strengths of our study. First, despite the retrospective nature of our study, our data are trustworthy and correct. The patient characteristics and mortality were consistent with those in well-designed prospective trials [4, 31, 32]. Second, no previous study has evaluated whether preoperative CRP and lymphocyte counts can predict mortality in patients with intertrochanteric fractures. Third, we present the mLCS, which is an easy-to-use and cost-effective prognostic indicator. mLCS can be easily assessed by using a routine biochemical test. The findings may help surgeons develop more effective strategies for preoperative care and improve survival rates.

There are some possible limitations to the study that should be acknowledged. First, this is a retrospective, observational study based on a database of electronic medical records of a particular institution. More extensive multicentre prospective randomized controlled studies are needed. Second, as an observational study, the analysis can only reveal a relationship, but not a causal relationship, between mLCS and mortality. Therefore, prospective studies with good designs must establish and verify a causal association between preoperative mLCS and 3-year mortality in these patients.

## Conclusions

In conclusion, the preoperative mLCS is a novel and promising indicator that can be used to simply and conveniently predict 3-year mortality in elderly patients with intertrochanteric fractures.

## Electronic supplementary material

Below is the link to the electronic supplementary material.

Supplementary Material 1

## Data Availability

The datasets used and/or analyzed during the current study are available from the corresponding author on reasonable request.

## Abbreviations

CRP: C-reactive protein
LCR: Lymphocyte C-reactive protein ratio
LCS: Lymphocyte C-reactive protein score
mLCS: Modified lymphocyte C-reactive protein score
DCA: Decision curve analysis
NRI: Net reclassification index
IDI: Integrated discrimination improvement index
HR: Hazard ratio
CI: Confidence interval
PLR: Platelet-to-lymphocyte ratio
NLR: Neutrophil-to-lymphocyte ratio
SII: Systemic immune-inflammation index
MAP: Mean arterial pressure
RBC: Red blood cell
ICU: Intensive care unit
SD: Standard deviation
IQR: Interquartile range
ASA: American Society of Anesthesiologists
CONUT: The Controlling Nutritional Status score
PNI: The Prognostic Nutritional Index score.
