# Supplementary material for: The modified lymphocyte C-reactive protein score is a promising indicator for predicting 3-year mortality in elderly patients with intertrochanteric fractures
Source: BMC Geriatr. 2023 Jul 12;23:432. doi: 10.1186/s12877-023-04065-z (PMC10339556; doi:10.1186/s12877-023-04065-z)
Supplement: Supplementary file 1 — Supplementary Material 1 [file 12877_2023_4065_MOESM1_ESM.docx]

**Supplementary materials**

# Supplementary Table 1. Long-term survival outcomes in different risk groups.

| **Cohorts** | **Time after surgery** | **LCS = 0** | **LCS = 1** | **LCS = 2** | ***P* value ^a^** | ***P* value ^b^** | ***P* value ^c^** |
| --- | --- | --- | --- | --- | --- | --- | --- |
| **LCS indicator** | 1-year OS rate (%) | 97.9% | 92.3% | 87.5% | 0.019 | <0.001 | 0.107 |
|  | 2-year OS rate (%) | 95.9% | 86.2% | 81.3% |  |  |  |
|  | 3-year OS rate (%) | 91.8% | 80.8% | 70.3% |  |  |  |
|  | **Time after surgery** | **mLCS = 0** | **mLCS = 1** | **mLCS = 2** | ***P* value ^a^** | ***P* value ^b^** | ***P* value ^c^** |
| **mLCS indicator** | 1-year OS rate (%) | 97.9% | 91.5% | 77.4% | 0.009 | <0.001 | 0.001 |
|  | 2-year OS rate (%) | 94.4% | 85.6% | 67.7% |  |  |  |
|  | 3-year OS rate (%) | 90.8% | 79.7% | 51.6% |  |  |  |

**Notes:** *P* value ^a^: comparison between 0 and 1; *P* value ^b^: comparison between 0 and 2; *P* value ^c^: comparison between 1 and 2.

**Abbreviation:** OS, overall survival; LCS, lymphocyte-CRP score; mLCS, modified lymphocyte CRP score; CRP, C-reactive protein.

# Supplementary Table 2. Long-term survival outcomes in different risk groups.

| **Cohorts** | **Time after surgery** | **LCS ≤ 1** | **LCS > 1** | ***P* value** |
| --- | --- | --- | --- | --- |
| **LCS indicator** | 1-year OS rate (%) | 94.7% | 87.5% | 0.004 |
|  | 2-year OS rate (%) | 89.9% | 81.3% |  |
|  | 3-year OS rate (%) | 85.5% | 70.3% |  |
|  | **Time after surgery** | **mLCS ≤ 1** | **mLCS > 1** | ***P* value** |
| **mLCS indicator** | 1-year OS rate (%) | 95.0% | 77.4% | <0.001 |
|  | 2-year OS rate (%) | 90.4% | 67.7% |  |
|  | 3-year OS rate (%) | 85.8% | 51.6% |  |

**Abbreviation:** OS, overall survival; LCS, lymphocyte-CRP score; mLCS, modified lymphocyte CRP score; CRP, C-reactive protein.

# Supplementary Table 3. Three-year mortality in different risk groups.

| **Groups** | **Survival, n (%)** | **Death, n (%)** | ***P* value ^a^** | ***P* value ^b^** | ***P* value ^c^** |
| --- | --- | --- | --- | --- | --- |
| **LCS = 0** | 89 (91.8%) | 8 (8.2%) | 0.020 | <0.001 | 0.102 |
| **LCS = 1** | 105 (80.8%) | 25 (19.2%) |  |  |  |
| **LCS = 2** | 45 (70.3%) | 19 (29.7%) |  |  |  |
| **mLCS = 0** | 129 (90.8%) | 13 (9.2%) | 0.010 | <0.001 | 0.002 |
| **mLCS = 1** | 94 (79.7%) | 24 (20.3%) |  |  |  |
| **mLCS = 2** | 16 (51.6%) | 15 (48.4%) |  |  |  |

**Notes:** *P* value ^a^: comparison between socre = 0 and socre = 1; *P* value ^b^: comparison between socre = 0 and socre = 2; *P* value ^c^: comparison between socre = 1 and socre = 2.

**Abbreviation:** LCS, lymphocyte-CRP score; mLCS, modified lymphocyte CRP score; CRP, C-reactive protein.

# Supplementary Table 4. Comparison of LCS and mLCS in predicting 3-year mortality in elderly patients with intertrochanteric fractures.

| **Indicators** | **C-statistics** | **95% CI** |
| --- | --- | --- |
| LCS | 0.644 | 0.564-0.725 |
| mLCS | 0.686 | 0.603-0.769 |

**Abbreviation:** CI, confidence interval; LCS, lymphocyte-CRP score; mLCS, modified lymphocyte CRP score.

# Supplementary Table 5. The NRI and IDI of mLCS compared to LCS in predicting 3-year mortality in elderly patients with intertrochanteric fractures.

|  | **Values** | **95% CI** |
| --- | --- | --- |
| NRI | 0.018 | -0.051-0.063 |
| IDI | 0.017 | -0.003-0.054 |

**Abbreviation:** NRI, net reclassification index; IDI, integrated discrimination improvement; CI, confidence interval; LCS, lymphocyte-CRP score; mLCS, modified lymphocyte CRP score.
